# Understanding the Impact of Child, Intervention, and Family Factors on Developmental Trajectories of Children with Hearing Loss at Preschool Age: Design of the AChild Study

**DOI:** 10.3390/jcm11061508

**Published:** 2022-03-09

**Authors:** Magdalena Dall, Sandra Kiblböck, Daiva Müllegger, Johannes Fellinger, Johannes Hofer, Ruth Kapplmüller, Sandra Breitwieser, Katharina Schossleitner, Christoph Weber, Ruth Zöhrer, Daniel Holzinger

**Affiliations:** 1 Research Institute for Developmental Medicine, Johannes Kepler University Linz, 4020 Linz, Austria; sandra.kiblboeck@bblinz.at (S.K.); daiva.muellegger@bblinz.at (D.M.); johannes.fellinger@bblinz.at (J.F.); johannes.hofer@bblinz.at (J.H.); ruth.kapplmueller@bblinz.at (R.K.); sandra.breitwieser@bblinz.at (S.B.); christoph.weber@jku.at (C.W.); daniel.holzinger@bblinz.at (D.H.); 2 Institut für Sinnes- und Sprachneurologie, Konventhospital Barmherzige Brüder Linz, 4020 Linz, Austria; katharina.schossleitner@bblinz.at; 3 Division of Social Psychiatry, Medical University of Vienna, 1090 Vienna, Austria; 4 Department of Pediatrics I, Innsbruck Medical University, 6020 Innsbruck, Austria; 5 MED-EL Medical Electronics Ges.m.b.H., 6020 Innsbruck, Austria; ruth.zoehrer@med-el.com; 6 Institut für Linguistik, Universität Graz, 8010 Graz, Austria

**Keywords:** pediatric hearing loss, epidemiological, family-centred early intervention, parent–child interaction, social communication

## Abstract

Children with hearing loss and their families represent a large variety with regard to their auditory, medical, psychological, and family resource characteristics. Despite recent advances, developmental outcomes are still below average, with a significant proportion of variety remaining unexplained. Furthermore, there is a lack of studies including the whole diversity of children with hearing loss. The AChild study (Austrian Children with Hearing Impairment—Longitudinal Databank) uses an epidemiological longitudinal design including all children living in Upper and Lower Austria with a permanent uni- or bilateral hearing loss below the age of 6 years, irrespective of additional disabilities, family language, and family resources. The demographic characteristics of the first 126 children enrolled in the study showed that about half of the children are either children with additional disabilities (31%) and/or children not growing up with the majority language (31.7%) that are usually excluded from comprehensive longitudinal studies. AChild aims for a characterization of the total population of young children with hearing loss including developmental outcomes. Another goal is the identification of early predictors of developmental trajectories and family outcomes. In addition to child-related predictors the examination of family–child transactions malleable by family-centred early intervention is of particular interest. The study is designed as participatory including parent representation atall stages. Measures have been chosen, following other large population-based studies in order to gain comparability and to ensure international data pooling.

## 1. Introduction

The World Health Organization (WHO) defines ‘disabling’ hearing loss in children as permanent hearing loss (HL) greater than 30 dB in the better ear. Globally, around 32 million children are affected by disabling HL [1], with greatly varying prevalence rates. In high-income countries, the rate of permanent bilateral severe to profound HL in newborns is 1.1 per 1000 [2]. Additionally, mild to moderate bilateral and unilateral HL affects 1–2 per 1000 newborns. While the research presented here is being conducted in Austria, a Western high-income country, it must be recognized that the great majority of children with disabling HL live in low- and middle-income countries (prevalence of disabling hearing loss in children in high-income countries = 0.5%; sub-Saharan Africa = 1.9%; and South Asia = 2.4%) [3].

There is great diversity in children who are deaf or hard-of-hearing (DHH), for instance, in the severity of HL, the affected region of the auditory pathway (e.g., sensorineural, conductive, and central) and the laterality of HL (unilateral/bilateral). In addition, a large variety of aetiologies can cause HL: around 60–70% of congenital HL is caused by genetic factors, 30% of which are syndromic [4]. Approximately 20–40% of DHH children have an additional disability [5]. Studies report a prevalence of intellectual disabilities in addition to HL in 13–26% of children, visual impairment in 4.4% and Autism Spectrum Disorder in around 3% [6,7,8]. DHH children also differ in the type of amplification used (hearing aids, cochlear implants, mixed, and no amplification).

### 1.1. Language and Psycho-Social and Family Outcomes

Newborn Hearing Screening (NHS), new technologies in hearing aids and cochlear implants, earlier fitting with hearing aids and cochlear implantation at a young age, a better understanding of the diverse aetiologies of HL and adaptation of intervention to the individual—and often complex—needs of the child and his/her family (including family-centred approaches) have led to large improvements in the lives of DHH children [9,10,11,12].

Despite these advances, many DHH children continue to lag behind their peers with typical hearing, particularly with regards to speech–language skills that, in turn, affect other critical dimensions of development. Some studies have reported language outcomes in DHH children without additional disabilities that are up to 1.5 standard deviations below those of typically hearing children of the same age [13,14,15,16,17]. The Outcomes of Children with Hearing Loss (OCHL) study showed that children with mild to severe HL fitted with hearing aids remained at risk for language delays [18]. In a follow-up study, children in second and fourth grade with mild to severe HL were examined. It was shown that children with moderate HL had oral language skills that were comparable to those of a control group with typical hearing, whereas the children with mild and moderate to severe HL did less well (almost one standard deviation lower). With regards to other academic skills (reading, writing, spelling, and mathematics), both groups of children with mild and moderate HL performed at a similar level as the control group. The children with moderate to severe HL were able to achieve comparable levels in writing and calculating to those of the control group [19].

A recent North American study comparing outcomes of two- to three-year-old children with mild to severe HL without additional disabilities with those of children with typical hearing demonstrated significantly lower scores in expressive and receptive language skills for the former. Notably, mean language scores of the children with HL were within the average range, but the proportion of children who were 1.5 SDs or more below the normative mean was significantly greater [20].

Poorer language skills can have a negative impact on children’s mental health and result in emotional and behavioural problems and difficulties with peer relationships. Highly elevated risk for psychosocial difficulties was reported by Theunissen et al. (2014) [21] and by Dammeyer (2010 [22]; factor 3.7). Other studies [20,23] found psychosocial development of young children (2–3 years of age) with HL on average to be within the typical range for children with normal hearing, but with a wider distribution.

The majority of studies investigating family resources have reported similar stress levels in mothers of children with HL and mothers of children with typical hearing [24,25,26]. Increased levels of parental stress have been found to be associated with slow communication–language development and child behavioural problems [24,25,27].

### 1.2. Child, Family and Intervention Factors That Affect Developmental Outcomes

The limited number of longitudinal studies available showed a diversity of child, family, and intervention variables that affect child development and, particularly, spoken language [12]. These factors can be categorized as either non-malleable (aetiology, and socioeconomic status) or malleable (audibility, age at provision with hearing technology, and family-child transactions). Identifying the malleable factors is particularly important in order to fully realize each child’s potential.

Among the child variables, audiological factors (e.g., degree of HL), audibility with hearing technology (hearing aid or CI benefit) and nonverbal cognition have been shown to be significantly associated with language outcomes [18,28,29]. A large population-based study in Australia showed that, aside from audiological factors, the presence of additional disabilities was the strongest factor that negatively affected expressive and receptive language development [30]. Additional disabilities have also been shown to significantly predict psychosocial and adaptive outcomes [21,22,23].

The importance of family environment to the language development of children with cochlear implants has also been demonstrated in a recent meta-analysis by Holzinger et al. (2020), in which the quality and quantity of parental linguistic input accounted for 31.7% of the variance in language outcome. Meta-analysis of family involvement/participation almost reached significance level and explained 14% of the variance. Maternal education has been found to significantly predict child language development [14,30], but not necessarily psychosocial outcomes [14,23,25,31]. Only recently have studies started to investigate the effect of maternal self-efficacy on child development. A study with 32 mothers of children with CIs showed a significant relationship between higher maternal self-ratings regarding their influence on the development of their child’s speech–language skills and their mean length of utterance (MLU) and use of parallel talk and expansion in their communication. This was subsequently related to children’s language outcomes [32]. A more recent study showed significant positive relationships between perceived maternal self-efficacy and language skills, adaptive behaviour, and early developmental abilities [25].

The often complex and reciprocal nature of relationships between environmental and child variables has been demonstrated for the influence of the family on the child and vice versa. Childhood HL impacts the family (e.g., coping with the diagnosis, disruption of natural parent–child interaction, dealing with a complex network of professionals, frustration about slow development, or behavioural issues) and often leads to elevated stress levels. In particular, child language and communication difficulties and behavioural problems have been found to be associated with higher parental stress levels [24,25,27,33]. Increased parental stress may in turn negatively impact parent–child interaction and, consequently, the child’s development [34,35]. As a result, the family environment needs to be investigated both as an outcome variable (e.g., parental stress, quality of life, and self-efficacy) and as a highly relevant predictor of child outcomes.

Among intervention variables predicting language outcomes early fitting with hearing aids and/or early cochlear implantation and timely access to family-centred early intervention as well as a higher intensity of early intervention haven been shown to predict language and functional communication outcomes [17,36,37,38]. International consensus based best practice of family-centred early intervention has been described and is supported by growing empirical evidence [39].

### 1.3. Rationale

Despite numerous improvements over the past decades, a significant proportion of children with HL continue to lag behind their hearing peers with respect to their speech-language skills, academic skills, and mental health. Individual differences are marked, and a high proportion of variability remains unexplained. This suggests the need for epidemiological longitudinal studies that include the full diversity of children and their families and for further exploration of factors and mechanisms that contribute to delayed or successful development. VicChild, a large population-based study in Victoria, Australia, with ongoing follow-up since 2012, is looking at various factors of child development. Especially during the children’s early years, the study focuses on parent reports, missing information from standardized assessments. Additionally, the sample is slightly skewed towards families with a higher SES. Also in Australia, before this study the Longitudinal Outcomes of Children with Hearing Impairment (LOCHI) collected prospective data on Australian DHH children [40]. Aside from these two studies, most other large studies on DHH children have been carried out in the US and are not epidemiological, but focus only on a specific group of children, such as the Outcomes of Children with Hearing Loss (OCHL) study with participants with mild to severe HL from three research sites in Iowa, North Carolina, and Nebraska [41] or the Childhood Development after Cochlear Implantation (CDaCI) study [42], which was limited to a sample of children with cochlear implants. Many studies face the problem that children from families with a higher socio-economic status are over-represented. Furthermore, children with additional disabilities, including complex syndromes, inborn errors of metabolism and neurodevelopmental disorders, and multilingual children are usually excluded from the study population. Excluding children because they struggle to complete the assessments may cause bias in relation to the prevalence of emotional and behavioural problems. This means that across existing studies a high proportion of children with HL is not included. In addition to these sampling biases, there is a complete lack of European epidemiological studies of DHH children and a strong focus on medical–audiological predictors of child development. Only in recent years has the family attracted research focus, and in an increasing number of studies the family of the DHH child is included as an influencing variable. The dynamic interaction between the family as an influential factor and the family as an outcome is often overlooked or not fully integrated within research studies; and even when family factors are considered, unresolved challenges in measuring family influence and outcome remain, as measuring and exploring the direction of the influence remains difficult.

The AChild (Austrian Children with Hearing Impairment—Longitudinal Databank) study is epidemiological and inclusive, involving the full diverse range of children with permanent HL, irrespective of family and cultural background. It is characterised by its multidimensional character (dependent and independent variables) and takes interaction effects into account in a high-income country with universal NHS and tracking program [43] and fully implemented medical, audiological, and family-centred interventions. This will make it possible to not only look at individual variables, that are already known to be of importance, such as early enrolment into intervention, early hearing aid or cochlear implant fitting, family–child interaction, and genetics separately, but to investigate their interaction and combined impact, without the exclusion of any child with HL. The epidemiological, longitudinal design will give insights into working mechanisms that effect developmental trajectories in children with diverse conditions. These are expected to inform clinical and early intervention practice. The study is designed to allow multicentre data pooling with other epidemiological longitudinal studies.

### 1.4. Objectives and Hypotheses

This study has four major objectives.

- Objective 1

Detailed characterisation of a total population of children with significant HL living in Upper and Lower Austria (baseline characteristics and outcomes) regarding their aetiological (including genotypical and phenotypical correlations), audiological, neurodevelopmental (nonverbal cognition, motor), psycho-social and linguistic/communicative characteristics, with particular focus on children with additional needs and describing family characteristics and outcomes.

- Hypothesis 1

There is a higher prevalence of comorbidities, such as intellectual disabilities, visual impairment, autism spectrum disorder, executive functioning and motor deficits, and health problems, in the group of children with HL than previously reported.

- Objective 2

Identification of early predictors (in children under the age of 2 years) of developmental trajectories with a focus on the search for malleable variables, such as family factors (parent–child interaction/communication, parental self-efficacy, and parental stress), audiological factors (audibility with well-fitted hearing technology, daily hearing aid/CI use, and age at hearing-aid fitting/cochlear implantation) that can be positively influenced by early intervention programs to support the families. Thus, we ultimately aim (i) to detect moderators of the relationship between child and family characteristics that are considered to be stable (e.g., degree of HL, nonverbal cognition, aetiology, and family SES) and functional outcomes of the child (e.g., language/social communication, and psycho-social) and of the family (e.g., family QoL, and parental stress) and (ii) to identify subgroups of children with HL by their developmental trajectories. Based on a better understanding of these interactions we expect to derive conclusions for the practice of multi-dimensional and multi-professional early intervention.

- Hypothesis 2

The following factors are expected to affect child linguistic/communicative and psychosocial outcomes:

Malleable factors:

- audiological factors (access to spoken language by means of early optimal fitting and constant use of hearing technology)
- access to signed language
- family factors (parental stress, family–child interaction, media use, and parental self-efficacy)
- family-centred intervention (mediated by family factors)

Non-malleable factors:

e. child HL (degree of HL)
f. child nonverbal cognition and additional disabilities/comorbidities
g. aetiology of HL (including genotype)
h. family factors (SES, parental education)

- Objective 3

To identify factors that influence family outcomes (quality of life, parental stress, and parental self-efficacy).

- Hypothesis 3

The following factors are expected to affect family outcomes:

(a) child linguistic and social communication development
(b) child behavioural problems
(c) child emotional problems
(d) child health
(e) quality of parent–child interaction (e.g., facilitative language strategies, and parental responsiveness)
(f) family-centred early intervention services
(g) non-malleable family resources (e.g., SES, and parental education)

- Objective 4

To evaluate existing and adapted measures (of spoken and signed language, social communication, psycho–social development, parent–child interaction, and other family measures) for accuracy and feasibility of use in the study population (children with HL and their families).

### 1.5. Data Resource Areas

There are approximately 30,000 births a year in Upper (14,757 births in 2020) and Lower (14,611 births in 2020) Austria [44]. The prevalence rate of significant unilateral and bilateral HL in newborns in Upper Austria is 1.75 per 1000 children [43]. In Austria, universal Newborn Hearing Screening (NHS) has been in place since 1995, and since 2003 it has been part of the regular well-baby check-up.

#### 1.5.1. Upper Austria

The “*Institut für Sinnes- und Sprachneurologie*” is part of the hospital Barmherzige Brüder Linz, and since 2009 it has offered “*FLIP*”(Family-centred Linz Intervention Program), the only early intervention program specifically for children who are DHH in Upper Austria. Since 2015, a tracking system has been implemented by the Institut für Sinnes- und Sprachneurologie in close collaboration with the Health Department of Upper Austria. NHS covers about 98% of newborns, and most infants with HL (81% of the 2017 birth cohort) are enrolled in family-centred early intervention before the age of 6 months [43], as recommended by international guidelines [45].

The single point of entry to FLIP ensures that almost every child (>95%) who is DHH is enrolled in the intervention program. The institute’s diagnostic centre “*Neurologisch-linguistische Ambulanz*” (NLA), situated in the Linz hospital, offers multi-professional assessment. Each regular diagnostic appointment includes assessments by a clinical linguist, a medical doctor (neuropediatrician) and an audiologist, and children older than 3 years are also seen by a clinical psychologist. This ensures a comprehensive assessment of all aspects of child development.

The scientific research for AChild is done at the Research Institute for Developmental Medicine, which is part of the Medical Faculty of Johannes Kepler University Linz. Many of the AChild team members are both hospital and university employees, which ensures close collaboration between diagnostics, intervention, and research.

The FLIP family-centred early-intervention program follows the 10 evidence-based principles described in the International Consensus Statement on Best Practices in Family-Centred Early Intervention [39]. The main focus is on meaningful engagement of the family in promoting effective everyday communication for their children; this is realized by parents and interventionists working closely together as partners. The goal is to promote parental competence and confidence to make informed decisions about their child’s development. The intervention takes place in the families’ homes once a week or every other week, depending on the developmental stage and needs of the child and family. The families are supported not only by the interventionists, but also by parent peers and deaf professionals who are also closely involved in the development and conception of the intervention program.

#### 1.5.2. Lower Austria

In contrast to Upper Austria, where FLIP has been implemented for 12 years, modern family-centred early intervention specifically for children with HL started only in 2020 in Lower Austria. The tracking system after the NHS is not yet as well established as in Upper Austria, and up to 2020 early intervention was offered by various providers of early intervention covering all kinds of developmental delays. Therefore, the mean age of children at enrolment in early intervention continues to be higher than in Upper Austria. A comparison of Lower Austrian data on the children and families transferred from the preceding intervention programs with Upper Austrian data on children (and their families) of the same age is expected to demonstrate effects of specialized family-centred early intervention.

## 2. Materials and Methods

### 2.1. Study Design

Based on a kind of unstructured multicohort design [46], this prospective study aims to establish a longitudinal epidemiological database. In this study children within the target age range from 0 to 5½ years are recruited across the duration of the study. Data is gathered at six child ages (9, 18, 27, 36, 48, and 66 months and at baseline (at enrolment in family centered early intervention (FCEI)). Thus, depending on the age a child enters the study, there are at least 1 and at a maximum seven measurement points for a child (i.e., a child that enters the study in the first study year at the age of nine months will ideally participate in all seven measurement points, and a 9-month-old child who enters the study in the second year will participate in six measurement points). Data collection started at the beginning of 2020, and follow-up and recruitment will continue until 2025 when the current funding will end. This study was approved by the ethics committee of the Konventhospital Barmherzige Brüder (EKB03-19) and registered at clinicaltrials.gov (NCT04317456).

In addition to the prospective data collection over five years, selected retrospective data extracted from the medical records of children who participated in the early intervention program between 2010 and 2020 will be added to the database, particularly data for time points before enrolment in AChild. Despite their incompleteness, the clinical data are expected to enrich the larger prospectively collected database. In order to guarantee reliability, data extraction of medical charts will be done by two raters independently.

From the beginning there has been a strong focus on the involvement of parents in the design and implementation of this study. The early interventionists and the parent peers make new parents aware of the study, and after detailed information has been given, all children whose parents give written consent are included in the study.

### 2.2. Participants

All pre-school children below the age of 6 years living in Upper and Lower Austria with bilateral or unilateral permanent HL with pure-tone thresholds in the better ear of ≥25 dB in at least two frequencies (0.5, 1, 2, and 4 kHz) can participate in the study. AChild includes children with HL and intellectual disability, psycho-social difficulties, additional sensory disorders, restricted motor skills, and learning disorders. Bilingual children are also included in the study sample. Excluded are children older than 6 years at the time of diagnosis, children of families who do not give consent to the use of the pseudo-anonymised data, and children with temporary conductive hearing loss. At any time of the study, parents can withdraw their written consent.

Every DHH child that was between the age 0 and 5½ years on 1 January 2020 was invited to participate in the study. Every child born after January 2020 is automatically invited to join the study. The baseline visit is the first contact between family and interventionist. It includes a medical history assessment by the medical doctor of the NLA team, where information about the socio-demographic background of the family and about the history of the child’s HL is gathered. All parents are informed about AChild, either at this first visit or at a later visit from the interventionist to manage the amount of new information presented at the first visit.

Annually, around 30–35 children living in Upper Austria are enrolled, and 15–20 new enrolments (the number is expected to increase continuously) are expected from Lower Austria (Table 1). Data collection takes place at seven time points that are linked to the chronological age of the child (baseline, 9, 18, 27, 36, 48, and 66 months).

In the period from 1 January 2020 to 30 April 2021, 126 children joined the study. Due to the study’s design, there were children in all six age groups. Table 1 shows the age distribution; most children were seen at the ages of 9, 27, and 36 months. A total of 28 children already had a second appointment within this period. The demographics of the children and their families are presented in Table 2. Around 30% of the children are multilingual, which is comparable to the proportion of multilingual families in the Upper Austrian population. Almost 12% of the parents are DHH themselves. The children started with family-centred early intervention at a median age of 5.5 months, with a large range between 0–64 months. Nearly 40% of the participants are female. Regarding the possible aetiology, 26.2% from the total sample were clinically characterised as syndromic, 57.9% as non-syndromic. Results of genetic testing are available for 52.4% of the children. And 17.5% are still awaiting the results of either whole exome testing, a hearing panel, or a connexin test. In the sample with genetic data already available, 18.2% have a syndromic aetiology, the evaluation of confirmed non-syndromic aetiologies is still ongoing, the largest confirmed group of non-syndromic HL are connexin-26 gene mutations (21.6%). For 16.7% the aetiology is uncertain after the genetic testing. 3.2% from the total sample had Cytomegalovirus (CMV) infection. Around one third of the children have a significant additional medical diagnosis.

The median age of diagnosis is 4 months, with a wide range of 0–64 months. And 20% of the total sample have unilateral HL, whereas about one third of unilateral hearing losses confirmed after newborn hearing screening are expected according to international data [47]. The median age of first hearing-aid fitting was 6 months, with a maximum of 73 months and 81% of the children using hearing aids. 20.7% of the children have unilateral or bilateral cochlear implants, with a median age at first implantation of 14.5 months. Regarding the type of HL, the great majority (81%) has a sensory neural hearing loss, and 8.7% have atresia. Concerning the degree of HL in the better ear, 5.6% have mild HL, 38.9% moderate HL, 14.3% severe HL, and 19% profound HL.

The participation rate within the described time frame is 86%, 18% of the nonparticipants have unilateral hearing loss.

### 2.3. Data Collection

We seek to have the appointments for clinical assessment as close to the study time points as possible, but there is a three-month window around the actual time point for the younger children (9, 18, 27, and 36 months) and a six-month window for the older children (48, and 66 months) within which they can be seen.

**Figure 1:**
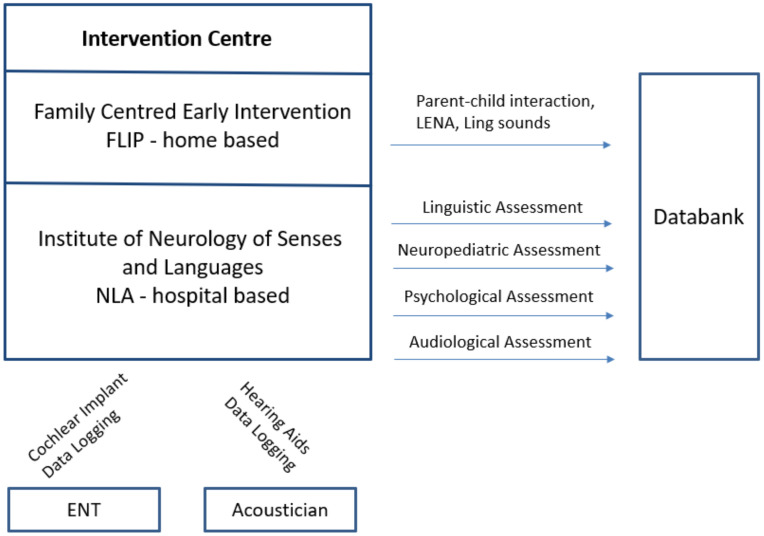
Data collection process.

The assessment is multi-professional. During the half-day diagnostic appointment, the child is seen by a medical doctor, a clinical linguist, and an audiologist. Starting when the child reaches the age of 3 years, a psychologist joins the diagnostic team (see Figure 1). All assessments in the clinic are direct, using standardised instruments. Before the appointment, the parents are requested (usually by their early interventionists) to complete questionnaires on the child’s speech, language and communication, emotional and behavioural aspects, parental stress, parental self-efficacy, and child and family quality of life. The early interventionists are available to support parents if the questionnaires are unclear to them, particularly in cases in which the family language is not German. There is also the option to include remote interpretation support for parents with insufficient German or English language skills to complete the questionnaires. In addition, early interventionists collect recordings of parent–child interaction. All results are discussed with the parents at the end of the clinical assessment, and a multi-professional report is written.

Considering the young age of the participants and their attention span, very young children are assessed in a playful setting with sufficient breaks. Being aware of the burden the parent reports present to the parents we have tried to keep the amount of work they have to complete before an appointment below 90 min while still including everything we consider vital to this study (see Table 3). All questionnaires are completed on paper. Within the year 2022, we will be evaluating whether it is preferable to complete questionnaires electronically and thus to enable parents to connect from home and complete them online.

In the AChild study, parents are regarded as the most important informants about their child’s development. Many questionnaires are parent-reported and help to gain a clearer picture of the child’s functional skills in his/her natural environment. Since assessing social communication skills of a child is particularly difficult in an artificial assessment situation, parent observations in the course of everyday life are essential. Collecting information from multiple sources—and, therefore, having multiple perspectives—contributes to a comprehensive picture.

Separate research teams were formed for medicine, linguistics/audiology, and psychology/psychiatry, and the examiners were trained in the assessments. Standard operating procedures (SOPs) were written for all assessments and questionnaires, including procedures to adapt the selection of measures to individual needs of children with severe developmental delays. Monthly project meetings provide opportunities for direct exchange between the person responsible for data management, the person responsible for data entry and examiners and therapists. This assures data quality and completeness. Completeness of parent questionnaires and procedures for collecting information on parent–child interaction are monitored by an early interventionist who is also part of the AChild research team.

In addition, in the morning of each direct AChild assessment day, the examiners meet to discuss questions regarding their test battery. This repeated direct interaction helps to keep fidelity high.

### 2.4. Measures

The questionnaires and assessments focus on the following dimensions: (a) hearing, (b) speech/language/communication, (c) nonverbal cognition, (d) psycho-social development, (e) medical factors, (f) intervention, and (g) family. All direct assessments used in the clinic are standardized measures. For language and communication there are no German assessments available that cover the entire age range from 9 months to 5½ years. Therefore, it was important to find contiguous measures that apply to the same construct in order for them to be comparable and to see continuous progress. Some questionnaires used in the study have not been translated into German and are not normed for German. These were therefore translated and back-translated, and for the time being the original American, British, or Australian norm data is used. One of the aims of the AChild study (objective 4) is to evaluate measures for validity and feasibility.

Table 4 includes all measures concerning child and family, indicating which are directly assessed and which are parent-reported.

AChild expects parent–child interaction to be one of the main variables that predict child social communication skills that relate to language acquisition, child mental health and a reduction of family stress. This highlights the importance of finding suitable measures for parent–child interaction. A recent systematic review has described the significant positive effects that joint attention, parental sensitivity and parental communication behaviours have on the language skills of children who are DHH [89]. AChild assesses parent–child interaction at 9, 18, and 27 months via video recordings made in the family homes. The aim is to observe parent–child interaction in a quasi-natural environment. This concept from the National Education Panel Study (NEPS) [90] makes it possible to compare the results with German norm data for children aged 7, 16, and 26 months. A 10-min semi-standardized playing situation between parent and child using selected age-appropriate toys is video-recorded by an early interventionist. The first three minutes involve playing with five familiar toys so that the child becomes comfortable with the situation. Subsequently, the standardized toys are placed within the child’s reach, and the parent is asked to play as they usually would. The evaluation comprises 10 dimensions relating to the parent (sensitivity to distress, sensitivity to non-distress, intrusiveness, detachment, domain-general stimulation, language stimulation, numeracy stimulation, positive regard for the child, negative regard for the child, and prevalence of affect) and five dimensions relating to the child (positive and negative moods, activity level, sustained attention, and sociability). Each dimension has five rating options, from not characteristic to very characteristic, plus the option to indicate that the dimension was not observed in the video sequence. A study has shown that the semi-standardized playing situation is comparable to a natural feeding and diaper change situation [91]. The NEPS study proved good inter-rater reliability and construct validity for this comparatively low-effort assessment. The first 10 videos in the AChild study were double rated by a psychologist and an interventionist. As soon as an inter-rater reliability of 95% had been reached, the interventionist continued single rating of the videos. Once the rater had gathered enough experience, rating a 10-min video took, at most, 15 min.

In addition to direct language assessments and various parent reports, the LENA (Language ENvironment Analysis) software is used to analyse the daily communicative interactions of the child within his/her natural environment. The early interventionist brings the LENA recording device to the families’ homes and instructs the parents. The child wears a T-shirt to which the device is attached, which records for 12 h. Parents are advised to do this on any day and to keep track of differences from “ordinary” days. The LENA recordings are done at 9, 18, and 27 months. Previous studies have shown that the number of turn-takings between child and parent in particular predicts future language outcomes [92]. Associations between number of words and conversational turns and socio-emotional skills one year later were also shown based on LENA [93]. Using the LENA devices in the AChild study is expected to provide further insights into influences of parental language and turn-taking on both the child’s social–communicative development and the family’s mental health. In addition, the feedback from the LENA evaluation can guide parents in their daily interactions with their child.

The LENA recordings will also be used to assess media use to complement a parent questionnaire about access to electronic devices and usage thereof. Previous studies with DHH children have shown that, unsurprisingly, children with more electronic media use had less time for direct interaction and showed poorer language outcomes [94]. The additional parent questionnaire on child media use has also been used in a total population study in Upper Austria, which can therefore be used as a reference group [87].

In addition to assessing parent–child interaction, a main aim of this study is to evaluate child social communication skills and the relationship with other domains of child development and family outcomes. Since social communication is not easily evaluated in unfamiliar situations, the Language Use Inventory (LUI) [66], a standardized parent-reported questionnaire is used. The questions are related to everyday life situations and are not limited to the first spoken language. Currently, the original norms are used, but our goal is to norm the questionnaire in a representative sample of children born in Upper Austria.

Unlike the majority of comprehensive longitudinal studies on children with HL—and this holds particularly true for European countries—we systematically include information on access to signed language and measures of signed language development in the study protocol despite a lack of standardized instruments for Austrian Sign Language. At every time of measurement parents report their use of Austrian Sign Language and/or any variety of simultaneous communication (simultaneous speaking and signing) with their child with HL by estimating the percentage of signed language as related to the total amount of child-centred parental language. Even though the parent questionnaire FRAKIS [58] (Fragebogen zur frühkindlichen Sprachentwicklung; Questionnaire on early language development), the German version of the MCDI [95], provides norms for spoken language only, we collect information on expressive signed vocabulary in addition to spoken vocabulary at the age of 18 and 27 months. To assess competencies in Austrian Sign Language beyond vocabulary we use two instruments that are still under development. A pilot study on a direct assessment of sign language reception based on the Reynell Developmental Language Scales and an adaptation of the subscale Language Reception of the Child Development Inventory (CDI) [96] (parent questionnaire) with a sample of 10 children of deaf adults (1;9–9;7 years of age) growing up with Austrian Sign Language as their family language showed promising results [65]. Correlations with chronological age and parent ratings of language development were high. Furthermore, results of direct and proxy measures were highly correlated with each other. By use of the LUI [66], child pragmatic skills rated by parents include the use of signed and spoken language.

As described earlier, we are interested in the reciprocal influences between family factors and child development. Numerous studies have linked quantitative and qualitative parental language use to better language skills in children [97], but evidence from twin studies indicates that there are also significant child-to-parent effects [98]. In addition to parental language input, we are particularly interested in the reciprocal influences of parental self-efficacy, family quality of life, and parental stress.

Parental self-efficacy also plays an important role in FCEI. The aim is not just to involve parents in the intervention, but to empower them by enhancing their competence and confidence [39]. For this purpose, a promising parent questionnaire—the SPISE-R (Scale of Parental Involvement and Self-Efficacy–Revised) [84] by Ambrose et al.—is used. This questionnaire asks parents of DHH children about their child’s use of the hearing device and how they would rate their knowledge and confidence in supporting their child [99].

The quality of life of the family is assessed with the PedsQL Family Impact Module [83]. In addition to the similar dimensions as the quality of life measure used for the children, this module also measures parent-reported family daily activities and family relationships.

To measure parental stress, the German version of the Parenting Stress Index (Eltern-Belastungs-Inventar) [82], a well-established parent questionnaire is used. This includes factors concerning the child that influence parental stress as well as parental resources/stress factors.

Audiological assessments are completed in an audiometric test booth by two pediatric audiologists. For testing air- and bone-conduction thresholds at 500, 1000, 2000, and 4000 Hz age-appropriate assessment techniques are used, including visual reinforcement audiometry, conditioned play audiometry, or conventional audiometry. For ear-specific thresholds preferably insert earphones (ER-3A), and circumaural headphones (HDA 200) are used. If participants do not tolerate testing with earphones or headphones, audiologists attempt to obtain soundfield thresholds. If the child stops participating in the assessment and testing cannot be completed, data from the child’s most recent audiogram are added to the database. Beside unaided thresholds, aided soundfield thresholds are measured. If possible, aided speech audiometry in quiet and noise are completed. To estimate the acoustic characteristics of the participants ears fitted with hearing-aids, Real-Ear-to-Coupler Difference (RECD) is measured. If RECD cannot be measured in the child’s ear due to lack of compliance, an age-related average RECD is used. HA verification is then simulated in the 2cc coupler. AURICAL^®^ FreeFit software (Natus Medical Denmark ApS, Taastrup, Denmark) is used for the calculation of aided SII, using the International Speech Test Signal presented at average (65 dB SPL) and soft (55 dB SPL) speech input levels.

The institute’s diagnostic centre does not provide hearing aid and cochlea implant fitting. Therefore, objective information on average amount of time the child uses his/her hearing technology (data logging) is collected in cooperation with local hearing aid acousticians and audiologists in cochlear implant centres who are in charge of fitting the devices. Hearing aid and cochlear implant use is also documented from parent report through a hearing aid questionnaire.

Regarding the genetic testing, there are two options. For children with the clinical impression of non-syndromic HL, a clinical exome sequencing (hearing panel) is offered, whereas for children with syndromic HL an analysis of the chromosomes and SNP array (primary) are offered. In this study, syndromic HL is defined as HL (uni- or bilateral) combined with ≥1 major malformation or with ≥2 minor malformations. Major Malformations are structural changes that have significant medical, social, or cosmetic consequences for the affected individual, and, typically, require medical intervention. Minor Malformations are structural changes without functional impact and significant malformations without known genetic association with HL. For the classification of Malformations we follow the EUROCAT documents (the European Network of population based registers for the epidemiological surveillance of congenital anomalies [100]).

### 2.5. Data Organization

All data is captured pseudonymously in REDCap (Research Electronic Data Capture) [101]. REDCap was chosen for its high security in data management and to facilitate international collaboration and data pooling. For standardized questionnaires and assessments, total raw scores, percentile scores and/or T-scores are entered, and for non-standardized questionnaires, data is entered at item level. At a later stage this will allow factor analysis to be performed when investigating the psychometric properties of the German translations. Three people were involved in setting up the database, who have good understanding of the questionnaires, and enter the data, which minimises mistakes due to inconsistent data entry. We are planning to roll-out electronic questionnaires through REDCap in the year 2022 and will additionally at the beginning offer paper-based questionnaires.

### 2.6. Statistics

Beside standard procedures that focus on the cross-sectional uni- and bivariate analyses and simple prospective predictions (e.g., predicting an outcome at age 66 months by variables gathered at age 9 months) the study design in conjunction with the study aims and hypotheses bring along several analytical issues and challenges that are briefly described here. Overall, we take a structural equation modeling (SEM) approach on analysing longitudinal data that offers a broad and flexible set of analytic procedures such as latent growth modeling and latent difference score modeling [102,103,104]. Moreover, a SEM framework allows to incorporate mediation and moderation hypotheses in longitudinal models and is compatible with growth mixture modeling (i.e., identifying different developmental trajectories) [104,105,106]. A specific challenge in longitudinal modelling arises when different tests must be used (depending on the age appropriateness of the tests) to assess a construct of interest at different ages and, thus, complicate the analysis of growth in a common metric. To achieve a common metric, it is possible to use linking approaches (e.g., use at least parts of the different tests simultaneously at a specific age) based on Item Response Theory (IRT) [107]. A next issue is associated with missing data. For example, if we are focusing on growth across the whole age range from 9 months to 66 months, there is missing data by design (i.e., only children who entered the study at the age 9 months in the first study year have data on all measurement time points) as well as missing data by unit- or item nonresponse (e.g., children who do not participate in a wave (unit) or parents who do not answer some questions (item)) and dropout. If missing data is due to a missing completely at random (MCAR) mechanism (i.e., missingness does not depend on any other observed or unobserved variables) or a missing at random (MAR) mechanism (i.e., missingness depends on observed variables) we can use state of the art procedures such as full information maximum likelihood estimation or multiple imputation [108].

### 2.7. Strengths of the AChild Study

AChild, with its epidemiological research design, is fully inclusive and therefore involves all children, including those (a) with additional disabilities, (b) who are multilingual, (c) who come from families with low SES, (d) with unilateral or mild hearing loss, (e) who use sign language or are bimodal, and (f) who are without amplification, who are excluded in most other studies. This will allow a comprehensive picture of all children with HL.

The implementation of the AChild study within the early intervention program gives the opportunity to have a close relation with the families through the interventionists. Parents thus receive regular feedback, which also helps with tracking the child’s development. An important aspect of this study is its participatory approach. Since the beginning of the study, a parent peer has been part of the study team and involved in the construction of the study design, bringing a parent’s point of view and feedback into the team.

At the “Institut für Sinnes- und Sprachneurologie”, diagnostic and therapeutic services are provided within one department. This ensures close collaboration between the two teams and improves communication flow.

The standardised multi-professional assessment in the clinic combined with the information given by parents and interventionists covers a large variety of child developmental domains including behaviour in natural settings. This is particularly important in areas of social communication where the “unnatural” assessment environment could bias the result, whereas parents can observe and assess their child’s skills in a rich variety of everyday social situations. Some of the methods for measuring parent–child interaction are standardised, such as analysis of the parent–child interaction, while some of the observations made by parents are unstandardised.

Collaborations with other epidemiological studies, such as VicChild, were planned from the beginning, and thus we focused particularly on data comparability in order to be able to collect sufficiently large epidemiological samples by data pooling that will then allow research, for example, into rare aetiologies or other smaller subgroups of children.

## 3. Discussion

The aim of this study is to gain extensive information on all relevant developmental dimensions of DHH children. The epidemiological study design ensures that all children are included and that insights are also gained on children with additional disabilities, multilingual children, children with unilateral HL, unlike previous studies that often had a bias towards including higher SES families or that outright excluded children with additional disabilities or multilingual children. Our approach requires extra time and involves interpreters or providing questionnaires in multiple languages. Furthermore, it is challenging to combine age-appropriate testing with testing that is appropriate to the individual child’s level of development. However, not including these children would mean that any new insights gained into the development of DHH children and their family environments would be informed by only a small subset of them.

To investigate the interaction between child, family, and intervention on growth trajectories, it is important to collect longitudinal data in order to see long-term influences and be able to investigate causal influences. This research should increase knowledge about optimally adjusted intervention for every child. Therefore, the strong focus is on malleable variables both regarding the child and the family. In order to make meaningful conclusions, a large enough sample size is important, considering the big variety of variables that are included. This will be achieved via three different approaches. Firstly, the longitudinal design enables the inclusion of children at all ages within the inclusion age range. Therefore, within a shorter period of time more children can participate. For longitudinal research questions, all children who have at least two data points can be included. Secondly, many of the instruments have been chosen in accordance to other longitudinal population-based studies, which will make data pooling across different countries possible, to answer research questions that relate to a small group of children or include a high number of independent variables in prediction models. Ideally, this is already considered in the conceptional phase and measures and time points are coordinated accordingly. In this case, legal data sharing agreements are required. Thirdly, multiple imputation will be used, so that missing data will not lead to the exclusion of entire data points.

One key aspect in modern research in a family-centred environment is the full involvement of the parents. They know their children best, and when it comes to aspects such as social communication, that cannot be tested exclusively in an assessment situation, their input is essential. In our experience, input from a parent peer, who articulates a parent’s perspective, wishes, and doubts, is particularly insightful. Especially in FCEI, we think that participatory research is the future, as much valuable input would otherwise be lost.

Considering the family not only as an influential variable, but also as an outcome variable that is related to child development, opens ways for research looking at reciprocal influences. To do this, we are using modern methods such as observation of semi-structured play situations between the child and the parent. The participatory approach enables the collection of information directly in the family. This requires trust from the families, contributing data from their personal family environment. The communication at eye level between the interventionists, the professionals and the parents is of utmost importance. The parents are contributing a large part to the study, which is recognized and highly appreciated. The relationship with the parents is based on continuous contact with the interventionists and professionals, which is, probably, one of the reasons for the high participation rate.

The multi-perspective approach of the study is possible because the diagnostic team, including medical, psychological, linguistic, and audiological professionals are working in the same institute as the intervention team including parent peers. Additionally, there is a close collaboration with outside partners such as acousticians, ENT-clinics, and educational infrastructure. The multi-perspective approach also allows for the multi-dimensional design. Child development has many different dimensions, and, since they are all intertwined, they must be investigated holistically while additionally considering the family. This requires good coordination.

Presently, there is still a lack of research on the use of language in everyday life and language and communication measurement early in life. Social communication is expected to impact language development, social cognition, peer-interaction, and mental health later in life. There is still a lack of tools to measure early social communication skills. Research on the effectiveness of early intervention is scarce. This is mostly due to a lack of control groups of children with HL not enrolled in early intervention. In addition, a comparison between intervention programs would require manualisations and fidelity of the programs. In this study we measure the effectiveness of family-centred intervention mainly indirectly through the family, that is, the skills and self-efficacy of the family. One of the main outcome measures is, whether the family could improve their transactions with their child during the time period of the intervention. This topic would also lend itself to qualitative research by outcomes-oriented parent interviews. It is important for future studies to address this research gap.

A further aspect that should be included in future research is the topic of cost-effectiveness. The plan for the upcoming years of the study is to include costs of intervention and long-term economic benefits.

Good data management in large longitudinal studies is crucial. Modern research will more and more rely on electronical data management as well as electronic questionnaires. This has the advantage of an increase of accuracy of data transfer. Nevertheless, it is important to adapt data collection to the needs of the families and take into consideration, that electronical questionnaires might hinder some families of participation.

### Challenges and Limitations

One of the greatest challenges is the fully inclusive design of the study. To include families with only little knowledge of German, extra effort in completing questionnaires, history taking or direct assessments of children—be it in the form of help from interpreters or from the interventionists—is required. Similar challenges may arise with parents and children who use sign language. Even though the interventionists and some of the clinicians are proficient in sign language, not all assessments can be fully adapted to visual communication.

With regards to the participation rate, we found that in comparison to international data on the rate of unilateral HL after newborn hearing screening children with unilateral HL seem to be underrepresented in our sample. This finding will require a careful look at the current tracking system with particular attention to unilateral HL.

A further challenge that we are facing concerns scheduling of the appointments for the diagnostics within the three- and six-month windows around the specified study time points. Multiple factors—such as the beginning of the COVID-19 pandemic, limited clinic slots, and challenges in arranging appointments that are convenient for the families—make it difficult to adhere to the set time frames.

## 4. Conclusions

Multiple factors, above all the quality and consistency of accessibility to language (audibility and/or access to signed language) and the quantity and quality of interactions with the family/care-givers are expected to influence communicative and psycho-social trajectories of children with HL. This study aims for the identification of factors, modifiable by intervention, that impact the development of children with HL with their highly varying needs and potentials. Modern research in the field of childhood HL requires integrative research including medical, audiological (hearing technology), and family factors in partnership with families and by use of methods that guarantee the inclusion of large enough volumes of data.

## Figures and Tables

**Table 1:** Expected participation of children (*n* = 126/*n* (estimated) = 306).

Age at Enrollment	Collection Wave					
	9 Months	18 Months	27 Months	36 Months	48 Months	66 Months
9 Months	40 (220)	11				
18 Months		9	4			
27 Months			21	9		
36 Months				28	4	
48 Months					15	
66 Months						14
Total at Each Age Level	40	20	25	37	19	14

Note: Projected ns in brackets. We expect at a minimum 45 children entering the study per year at age 9 months.

**Table 2:** Baseline characteristics of children who entered in the period 1 January 2020–30 April 2021.

		N = 126
Family Characteristics		
	Hearing status parent, DHH parent, *n* (%)	15 (11.9)
	Maternal education, *n* (%)	
	Compulsory school, *n* (%)	8 (6.3)
	Apprenticeship, *n* (%)	14 (11.1)
	Technical school with diploma, *n* (%)	14 (11.1)
	University, *n* (%)	15 (11.9)
	Multilingual yes, *n* (%)	39 (31)
Family-Centred Early Intervention		
	Age at entry into early intervention, m (SD)	12.63 (15.91)
	Age at entry into early intervention, median (range)	5.50 (0–64)
Child characteristics		
	Sex female, *n* (%)	49 (38.9)
	Clinical classification	
	Syndromic, *n* (%)	33 (26.2)
	Non-syndromic, *n* (%)	73 (57.9)
	Genetic testing	
	Available, *n* (%)	66 (52.4)
	Waiting for final report, *n* (%)	22 (17.5)
	Confirmed etiologies	
	CMV, *n* (%)	4 (3.2)
	Non-syndromic	
	Connexin, *n* (%)	18 (14.3)
	Further non-syndromic	pending
	Non-syndromic mimics, *n* (%)	3 (2.4)
	Syndromic etiologies	
	Trisomie 21, *n* (%)	4 (3.2)
	Other (CHARGE syndrome, Cornelia-de-Lange syndrome, Goldenhar syndrome, micro deletion syndrome 6q12, syndromal tegmental cap dysplasia, Leopard syndrome, Waardenburg syndrome type 2A), *n* (%)	8 (6.4)
	Uncertain after genetic testing, *n* (%)	21 (16.7)
	Additional diagnoses, *n* (%)	40 (31.7)
	Autism Spectrum Disorder, *n* (%)	2 (1.6)
	Visual impairment, *n* (%)	15 (11.9)
	Inborn errors of metabolism, *n* (%)	1 (0.8)
	Cognitive development below average (≥1 SD below mean), *n* (%)	15 (12.4)
	Global developmental delay (>2 SD below mean), *n* (%)	15 (12.4)
Hearing loss characteristics		
	Age at hearing loss diagnosis in months, mean (SD)	8.94 (13.3)
	Age at hearing loss diagnosis in months, median (range)	4 (0–64)
	Hearing loss laterality, *n* (%)	
	Bilateral	101 (80.2)
	Unilateral	25 (19.8)
	No Hearing loss right/left	10 (7.9)/15 (11.9)
	Hearing Aid	
	Age of first fitting in months, mean (SD)	12.03 (14.2)
	Age of first fitting in months, median (range)	6.00 (1–73)
	Current use bilateral, *n* (%)	81 (64.3)
	Current use unilateral, *n* (%)	21 (16.7)
	Cochlear Implant	
	Bilateral, *n* (%)	22 (17.5)
	Age of 1st implant in months, mean (SD)	18.23 (7.75)
	Age of 1st implant in months, median (range)	14.5 (10–37)
	Age of 2nd implant in months, mean (SD)	18.23 (7.75)
	Age of 2nd implant in months, median (range)	14.5 (10–37)
	Unilateral, *n* (%)	4 (3.2)
	Age of implant in months, mean (SD)	29 (15.8)
	Age of implant in months, median (range)	26.5 (15–48)
	Concurrent hearing aid and cochlear implant use, *n* (%)	3 (2.4)
	Type of Hearing loss, *n* (%)	
	Sensorineural	102 (81)
	Auditory Neuropathy Spectrum disorder	3 (2.4)
	Atresia	11 (8.7)
	Permanent conductive	1 (0.8)
	Other	6 (4.8)
	Degree of hearing loss in the better ear (bilateral), *n* (%)	
	Mild (26–40 dB)	7 (5.6)
	Moderate (41–70)	49 (38.9)
	Severe (71–90)	18 (14.3)
	Profound (>91)	24 (19)
	Degree of hearing loss (unilateral), *n* (%)	
	Mild (26–40 dB)	2 (8.0)
	Moderate (41–70)	5 (20.0)
	Severe (71–90)	7 (28.0)
	Profound (>91)	4 (16.0)
	Unknown (Atresia)	7 (28.0)

Note: CHARGE = Coloboma of the eye, Heart defects, Atresia of the choanae, restriction of growth and development, and Ear abnormalities and deafness; CMV = cytomegalovirus; and DHH = deaf and hard of hearing.

**Table 3:** Time burden of assessments and questionnaires for parents and child.

	Baseline	9 Months	18 Months	27 Months	36 Months	48 Months	66 Months
Parents at home	6 min	35 min	75 min	60 min	70 min	40 min	75 min
Clinical assessment child	85 min	85 min	85 min	80 min	105 min	95 min	120 min
Interview parents	10 min	30 min	20 min	20 min	20 min	5 min	25 min

**Table 4:** Measures and Instruments.

					Months					
Construct	C	F	Measure	Baseline	9	18	27	36	48	66
*Hearing*										
Objective hearing threshold	x		ABR, ASSR	●						
Hearing threshold	x		PTA (0.5, 1, 2, 4 kHz)	●	●	●	●	●	●	●
Aided hearing threshold	x		PTA (0.5, 1, 2, 4 kHz)	●	●	●	●	●	●	●
Hearing aid/CI use	x		Walker [48]		●	●	●	●	●	●
			Data logging		●	●	●	●	●	●
*Speech perception*										
Hearing performance	x		Little Ears [49]		●	●				
			PEACH [50]			●	●			
Audibility	x		Speech intelligibility index (SII)		●	●	●	●		
Sound identification	x		Ling-sounds		●	●	●			
Word identification	x		Mainzer Kindersprachtest [51]					●	●	
			Göttinger Kindersprachtest [52]						●	●
			Freiburger Sprachverständnistest [53]							●
*Speech production*										
Intelligibility	x		Intelligibility in Context Scale (ICS) [54]				●			●
Vocal Development	x		Little Ears Speech Production [55]		●	●				
*Language/Communication*										
Use of gestures	x		ACDI Early Gestures [56]		●					
Symbolic behavior	x		CSBS [57]		●	●				
Vocabulary expressive	x		FRAKIS [58]			●	●			
			SETK-2 subtest word production [59]				●	●		
			SET 3-5 picture naming [60]					●	●	●
Vocabulary receptive	x		SETK-2 word comprehension [59]				●			
			PPVT-4 [61]							●
Grammar expressive	x		Subject-Verb agreement and Verb 2nd-word order				●	●		
			LogikS Grammar expressive [62]						●	●
Language receptive	x		PLS-5 [63]			●	●			
			SETK-2 sentence comprehension [59]				●	●		
			TROG-D [64]					●	●	●
			RDLS sign language use [65]				●		●	●
			CDI for language other than German			●		●		●
			CDI for sign language [65]				●			●
Social communication	x		LUI [66]			●	●	●		
			CCC-2 [67]							●
			FOCUS [68]						●	●
*Cognition*										
Non-verbal development	x		Bayley [69]		●	●	●			
			SON-R 2 ½-7 [70]					●	●	●
Executive functioning	x		BRIEF-preschool [71]					●		●
Adaptive skills	x		Vineland-3 [72]		●	●	●	●	●	●
Visual successive memory and attention	x		KNOX cube test [73]					●	●	●
Phonological working memory	x		Mottier test [74]					●	●	●
*Psychosocial*										
Quality of life	x		PedsQL 1–12 months [75]		●					
			PedsQL 13–24 months [76]			●				
			PedsQL 2–4 years [77]				●	●	●	
			AUQUEI [78]							●
Emotional and behavioral problems	x		CBCL [79]				●			●
Mental health	x		SDQ [80]				●	●	●	●
Coping		x	Family Stress and Coping Interview [81]			●			●	
*Medical*										
Genetics	x									
Motor skills	x		Neurological Status	●	●	●	●	●	●	●
Health status	x		Neurological Status	●	●	●	●	●	●	●
Intervention										
*Family*										
Socio-economic data		x	SES questionnaire		●					●
Parental stress		x	Parental Stress Index [82]					●		●
Family quality of life		x	PedsQL Family [83]		●	●		●		
Family involvement and self-efficacy		x	SPISE-R [84]	●			●			●
Parent-child communication		x	LENA [85]		●	●	●			
Parent-child interaction		x	EKIE [86]		●	●	●			
Media use	x		Media use questionnaire [87]					●		●
Reading time	x		Dialogic book reading questionnaire [88]			●	●	●	●	

● direct assessment; ● parent-reported. Note. ABR = Auditory Brainstem Response; ACDI = Austrian Communicative Development Inventory; ASSR = Auditory Steady-State Response; AUQUEI = Autoquestionnaire de qualité de vie enfant imagé; Bayley = Bayley Scales of Infant and Toddler Development; BRIEF-P = Behavior Rating Inventory of Executive Function-Preschool Version; CBCL = Child Behavior Checklist; CCC-2 = Children’s Communication Checklist; CDI = Child Development Inventory; CSBS = Communication and Symbolic Behavior Scales; FOCUS = Fokus auf den Erfolg der Kommunikation für Kinder unter 6 Jahren; FRAKIS = Fragebogen zur frühkindlichen Sprachentwicklung; ICS = Intelligibility in Context Scale; LENA = Language Environment Analysis; LUI = Language Use Inventory; PEACH = Parent’s Evaluation of Aural/Oral Performance of Children; PLS-5 = Preschool language scale fifth edition; PPVT-4 = Peabody Picture Vocabulary Test; PTA = Pure Tone Average; RDLS = Reynell Developmental Language Scales; SDQ = Strength and Difficulties Questionnaire; SET 3-5 = Sprachstandserhebungstest für Kinder im Alter zwischen 3 und 5 Jahren; SETK-2 = Sprachentwicklungstest für zweijährige Kinder; SII = Speech Intelligibility Index; SON-R 2½-7 = SON-R 2½-7 Non-verbal intelligence test; SPISE = Scale of Parental Involvement and Self-Efficacy—Revised; TROG-D = Test zur Überprüfung des Grammatikverständnisses.

## Data Availability

The data presented in this study are available on request from the corresponding author. The data are not publicly available due to data protection issues.
